# Genome-wide co-occupancy of AML1-ETO and N-CoR defines the t(8;21) AML signature in leukemic cells

**DOI:** 10.1186/s12864-015-1445-0

**Published:** 2015-04-17

**Authors:** Daniel J Trombly, Troy W Whitfield, Srivatsan Padmanabhan, Jonathan AR Gordon, Jane B Lian, Andre J van Wijnen, Sayyed K Zaidi, Janet L Stein, Gary S Stein

**Affiliations:** Department of Biochemistry and Vermont Cancer Center, University of Vermont College of Medicine, 89 Beaumont Avenue, Burlington, VT 05405 USA; Department of Cell and Developmental Biology, University of Massachusetts Medical School, 55 Lake Avenue North, Worcester, MA 01655 USA; Program in Bioinformatics and Integrative Biology, University of Massachusetts Medical School, 55 Lake Avenue North, Worcester, MA 01655 USA; Current address: Biomedical Sciences, Mayo Clinic, 200 First Street SW, Rochester, MN 55905 USA

**Keywords:** AML, Runx1, t(8;21), Chromosomal translocation, Myeloid cell differentiation, Epigenetic control

## Abstract

**Background:**

Many leukemias result from chromosomal rearrangements. The t(8;21) chromosomal translocation produces AML1-ETO, an oncogenic fusion protein that compromises the function of AML1, a transcription factor critical for myeloid cell differentiation. Because of the pressing need for new therapies in the treatment of acute myleoid leukemia, we investigated the genome-wide occupancy of AML1-ETO in leukemic cells to discover novel regulatory mechanisms involving AML-ETO bound genes.

**Results:**

We report the co-localization of AML1-ETO with the N-CoR co-repressor to be primarily on genomic regions distal to transcriptional start sites (TSSs). These regions exhibit over-representation of the motif for PU.1, a key hematopoietic regulator and member of the ETS family of transcription factors. A significant discovery of our study is that genes co-occupied by AML1-ETO and N-CoR (e.g., *TYROBP* and *LAPTM5*) are associated with the leukemic phenotype, as determined by analyses of gene ontology and by the observation that these genes are predominantly up-regulated upon AML1-ETO depletion. In contrast, the AML1-ETO/p300 gene network is less responsive to AML1-ETO depletion and less associated with the differentiation block characteristic of leukemic cells. Furthermore, a substantial fraction of AML1-ETO/p300 co-localization occurs near TSSs in promoter regions associated with transcriptionally active loci.

**Conclusions:**

Our findings establish a novel and dominant t(8;21) AML leukemia signature characterized by occupancy of AML1-ETO/N-CoR at promoter-distal genomic regions enriched in motifs for myeloid differentiation factors, thus providing mechanistic insight into the leukemic phenotype.

**Electronic supplementary material:**

The online version of this article (doi:10.1186/s12864-015-1445-0) contains supplementary material, which is available to authorized users.

## Background

Runx1, also known as AML1, is a frequent target of chromosomal translocations in myeloid progenitor cells [1]. The t(8;21) chromosomal translocation accounts for approximately 15% of acute myeloid leukemia (AML) cases and produces the AML1-ETO fusion protein [2]. AML1-ETO is comprised of the AML1 N-terminus, containing the conserved DNA-binding runt homology domain (RHD), fused with almost the entire eight twenty-one (ETO) protein [3]. ETO contains four conserved *nervy* homology regions (NHR) that bind different transcriptional repressive complexes including histone deacetylases and the silencing mediator of retinoic acid and thyroid hormone receptor (SMRT) complex [4]. All four NHRs are retained in AML1-ETO, and early reports demonstrated that the fusion protein represses the transcription of AML1 target genes important for myeloid differentiation [5]. This repression is mediated, in part, by interactions between AML1-ETO and the nuclear co-repressor protein (N-CoR) [6,7]. Recruitment of histone deacetylases (HDACs) by AML1-ETO and N-CoR leads to a loss of histone modifications associated with transcriptional activation (e.g., H3K9ac), whereas blockade of HDAC activity results in partial differentiation of leukemic cells [8-10]. In addition, the acquisition of repressive histone modification marks, including H3K27me3, is believed to serve as an epigenetic mechanism for AML1-ETO mediated gene repression [11,12].

The repressive activity of AML1-ETO does not represent its full range of functions. The fusion protein has also been shown to activate genes [13-15], and a mechanism for this transcriptional activation involving AML1-ETO and p300 interactions has recently been described [16]. AML1-ETO affects the function of microRNAs (miRs [15,17]), DNA repair proteins [18], and growth factors in myeloid progenitor cells [19]. The fusion protein also plays a role in epigenetic-controlled cell growth via interactions with rDNA repeats [20]. In addition to regulating gene expression directly, AML1-ETO interferes with the transcriptional activities of molecules important for myeloid cell differentiation via protein-protein interactions and acts as an organizer of cofactor exchange [21-23]. Taken together, these studies showed that AML1-ETO acts as a transcriptional regulator and modifies transcription factor activity via differential co-factor recruitment, properties that maintain the oncogenic character of t(8;21) leukemic cells.

Recently, genome-wide binding of AML1-ETO, AML1, and p300 has been determined in leukemic cells [24-26]. These studies have shown the following: global AML1 and AML1-ETO binding sites largely overlap [24], ETS-family proteins recruit AML1-ETO [27], and that PU.1, a master regulator of myeloid cell differentiation, is part of the t(8;21) core transcriptional network. AML1-ETO and the coactivator p300 co-occupy hypoacetylated genomic loci in leukemic cells [26], yet the relevance of this phenomenon to t(8;21) leukemia is not well-understood. In addtion, global interactions between AML1-ETO and N-CoR have not been studied. To clarify these issues, we employed chromatin immunoprecipitation with high-throughput sequencing (ChIP-seq [28]) and determined genome-wide sites of enrichment for AML1, AML1-ETO, N-CoR, and p300 in Kasumi-1 cells, a model system for t(8;21) leukemia [29]. ChIP-seq libraries for histone modifications associated with transcriptional activation (H3K4me3) and repression (H3K27me3) were also generated to assess whether epigenetic mechanisms account for the differentiation arrest phenotype in Kasumi-1 cells.

From our genome-wide analysis of AML1/AML1-ETO occupancy, we have identified and described a phenotypically relevant subset of putative regulatory sequences. These sequences are characterized by abundant N-CoR co-occupancy, relative to other AML1/AML1-ETO-bound sequences, and a significant enrichment in PU.1 motifs. Moreover, using publicly available gene expression data [24,30], we show by *in silico* analysis that genes associated with the AML1-ETO/N-CoR co-occupancy signature display significantly greater recovery of expression upon reduction of AML1-ETO mRNA levels than do other AML1-ETO-bound genes. AML1-ETO/N-CoR co-occupied genomic loci tended to be distal from transcriptional start sites (TSSs) and showed little enrichment in the H3K4me3 histone modification. Finally, gene ontology analysis of genomic regions associated with AML1-ETO/N-CoR enrichment was more relevant to the differentiation block exhibited by Kasumi-1 cells compared to those regions enriched in AML1-ETO/p300. Thus, although AML1-ETO both represses and activates genes at the single-gene level [31], our genome-wide data show that AML1-ETO predominatly acts as a repressor. Our studies provide a new understanding of the global mechanisms that regulate the t(8;21) leukemic phenotype.

## Results

### AML1-ETO associates preferentially with the co-repressor N-CoR

**Figure 1:**
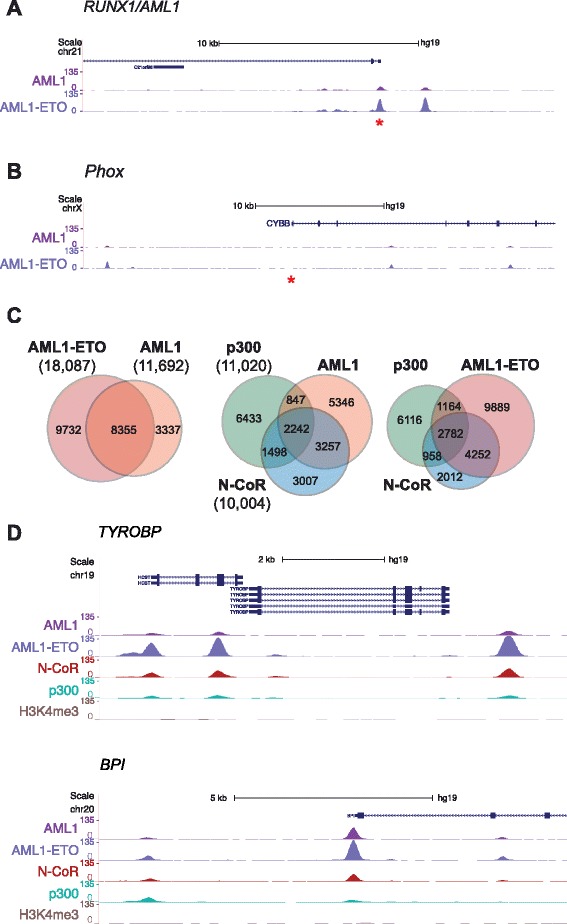
ChIP-seq analysis reveals global protein co-occupancy in Kasumi-1 cells. Tag density plots of normalized ChIP-seq data (combined biological replicates) showing AML1 and AML1-ETO occupancy at Runx1P1 promoter **(A)** and negative control Phox **(B)** loci. Tags were normalized to 10^7^ reads. Images were taken from the UCSC Genome Browser [63]. Asterisks indicate the positions of hRunx1P1 and hPhox regions tested in ChIP-PCR studies. **(C)** Two-way and three-way Venn diagrams displaying co-occupancy and unique regions among AML1, AML1-ETO, N-CoR, and p300 libraries. All peaks were generated by MACS using a p < 10^−20^ significance cutoff. Total peak numbers are displayed in parentheses. **(D)** Tag density plots displaying enrichment of AML1-ETO and N-CoR at regions corresponding to known fusion protein target genes that are repressed in Kasumi-1 cells.

ChIP-seq studies were performed to identify AML1 and AML1-ETO binding regions globally in the Kasumi-1 cell genome. In addition, ChIP libraries for molecular indicators of transcriptional activation (p300 and H3K4me3) and transcriptional repression (N-CoR and H3K27me3) were generated. Prior to library preparation, antibodies were validated through western blot and ChIP-PCR experiments (Additional file 1: Figure S1 and Additional file 2: Figure S2). For example, a known AML1 binding region within the Runx1P1 promoter [32] was significantly enriched in AML1-ETO ChIP samples compared to IgG control samples (Additional file 2: Figure S2A). This finding was confirmed in our ChIP-seq data, as sequence tag density showed strong AML1-ETO binding at the Runx1P1 site and negligible binding at a negative control Phox region (Figure 1A and B [33]). ChIP-seq data for AML1 yielded a similar binding profile at the Runx1 promoter (Figure 1A). An AML1 antibody that recognizes the C-terminus of Runx1, and therefore does not pull down AML1-ETO, was used in ChIP-seq library preparations.

Model-based analysis of ChIP-seq data (MACS [34]) was used to identify protein binding or enrichment regions (peaks) for histone modifications, AML1-ETO and associated co-regulators in the Kasumi-1 genome (Additional file 3: Table S1). AML1-ETO was enriched at genes that are known to be regulated by the fusion protein [15,17,24,30], underscoring the quality of our ChIP-seq data (Figure 1D and Additional file 4: Figure S3). Approximately 71% of AML1 peaks overlapped with those of AML1-ETO (Figure 1C). This result was expected, as AML1-ETO retains the DNA-binding RHD found in wild-type Runx1. Interestingly, 39% of AML1-ETO peaks overlap those of N-CoR, whereas only 22% of AML1-ETO peaks overlap those of p300 (Figure 1C). A similar profile was observed when comparing the co-occupancy of AML1 with the two co-regulators. These findings are consistent with a correlation analysis of genomic occupancy from ChIP-seq measurements (Additional file 5: Figure S4), which shows that AML1-ETO and N-CoR signals are more correlated with one another than either is with any of the other assayed DNA-binding proteins, co-regulators or epigenomic modifications. A closer analysis of selected genes that are related to the leukemia phenotype and are deregulated upon siRNA-mediated AML1-ETO depletion [24,30] (please see below) further confirms an AML1-ETO/N-CoR dominant co-occupancy pattern that may regulate the leukemic phenotype at the genome-wide level (Figure 1D).

**Figure 2:**
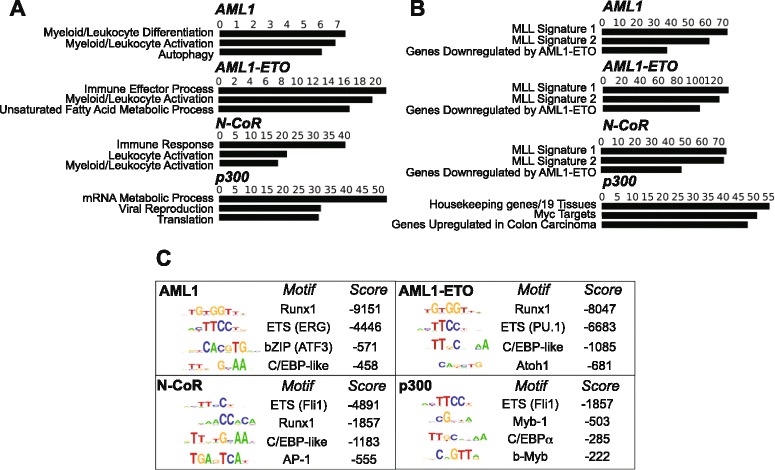
AML1-ETO and N-CoR peaks relate to similar gene ontology terms and motifs discovered *de novo*. (**A**) Gene ontology was used to infer biologically relevant pathways among ChIP-seq libraries using highly-ranked peaks (p < 10^−75^ from MACS). Shown are top-ranked gene ontology terms within Biological Process **(A)** and Molecular Signatures Database Perturbation **(B)** [64]. Values on column plots represent –log_10_(binomial p-value) computed using GREAT (version 2.0.2) [35] with the default association rules. Note: some ontology term names were shortened. **(C)** HOMER *de novo* motif analysis [54] of AML1, AML1-ETO, N-CoR, and p300 libraries. Displayed are the top-ranked motifs for each library with log(p-value) scores. Genomic loci occupied by AML1, the fusion protein and N-CoR were enriched in the Runx1 motif. Candidate transcription factors that harbor these motifs (listed in the ranked list of motif results) are shown in parentheses.

To evaluate the gene-sets that are likely to be perturbed by the binding of AML1-ETO and its cofactors, we analyzed gene ontology terms. ChIP-seq peaks with high statistical significance (p < 10^−75^) from each library were analyzed using the Genomic Regions Enrichment of Annotations Tool (GREAT [35]). Enriched gene ontology terms germane to molecular signatures of hematopoietic and leukemic cells were similar among the AML1, AML1-ETO and N-CoR datasets, but not that of p300 (Figure 2A and B). Importantly, ontology terms derived from genomic loci co-occupied by AML1-ETO and N-CoR were more relevant to myeloid leukemia than those from regions exclusively co-occupied by AML1-ETO and p300 (Additional file 6: Figure S5). Ontology terms derived from regions that display enrichments of AML1 with p300/N-CoR and AML1-ETO with p300/N-CoR resemble those of the fusion protein and N-CoR (Additional file 6: Figure S5). Thus, AML1-ETO preferentially associates with N-CoR compared to p300, and genes represented by these sites reflect the leukemic phenotype of Kasumi-1 cells.

Transcription factors act in a combinatorial fashion to regulate gene expression in hematopoietic progenitor cells [36]. A similar situation likely occurs in Kasumi-1 cells, where interactions between AML1-ETO and distinct co-regulatory proteins at hematopoietic genes may be necessary to maintain differentiation arrest [25,27]. In our genome-wide study, *de novo* motif discovery revealed that Runx1 and ETS family motifs were most commonly associated with AML1 and AML1-ETO peaks (Figure 2C). ETS family motifs (please see the Discussion section for candidate ETS motifs) were also enriched in sites of N-CoR and p300 occupancy (Figure 2C). Taken together, our data suggest that the differentiation arrest in Kasumi-1 cells is caused by putative global AML1-ETO/ETS interactions and through the repressive activity of the fusion protein on AML1-mediated transcription.

### PU.1 motifs are significantly associated with regions of elevated AML1-ETO/N-CoR occupancy and low H3K4me3 enrichment

**Figure 3:**
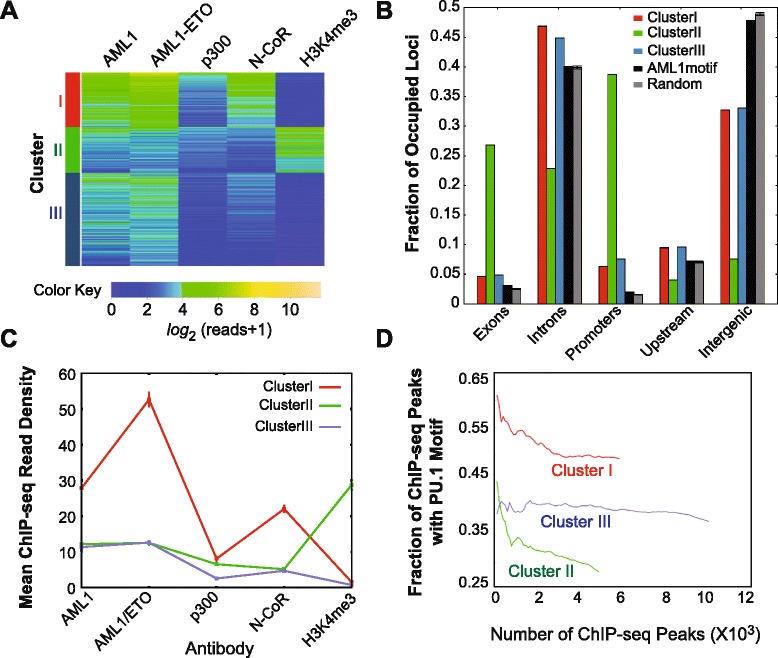
Classification of AML1/AML1-ETO genomic localization using co-factors. **(A)** Heatmap of significant (p < 10^−20^ from MACS) AML1/AML1-ETO ChIP-seq peaks clustered using k-means (k = 3) with normalized mean (over summit +/- 50 bp regions) read densities for AML1, AML1-ETO, N-CoR, p300 and the transcription initiation-associated histone modification H3K4me3. Following the color key, lowest read densities are represented in dark blue, whereas highest signals are in tan. **(B)** Distribution of clustered AML1/AML1-ETO loci among five classes of genomic elements. The y-axis represents the fraction of occupied loci among the each of the three clusters. **(C)** Profile of within-cluster averaged AML1, AML1-ETO, and co-regulatory protein enrichments. **(D)** Plot showing the fraction of ChIP-seq peaks containing the PU.1 motif as a function of total peak numbers for each cluster.

To characterize further the relationship between AML1, AML1-ETO, the co-regulators p300 and N-CoR, and epigenetic signatures, the mean tag densities for these datasets over regions (100 bp, centered on the summits of ChIP-seq peaks) bound by AML1 and/or AML1-ETO were clustered using k-means (Figure 3A). ChIP-seq enrichments for H3K27me3 showed little correspondence with signals from all other ChIP libraries (data not shown), yet this repressive mark was associated with some AML1-ETO regulated genes including CXCR4 and HCK (Additional file 7: Figure S6). At genomic regions where levels of AML1-ETO and N-CoR enrichment were highest (Cluster I), enrichment of the activating mark H3K4me3 was low (Figure 3A and C). This anti-correlation is expected on the basis that only a small fraction (6.3%) of the AML1/AML1-ETO-bound loci from Cluster I are found at promoters of the UCSC known genes (Figure 3B [37]), whereas H3K4me3 enrichments are expected to be elevated at actively transcribed genes [38]. In contrast, Cluster II was characterized by an inverse pattern, where occupancy of AML1-ETO and N-CoR was lower (Figure 3C) but statistically significant (p < 10^−20^ for AML1-ETO and/or AML1 enrichment) and enrichment of H3K4me3 was high. The mean occupancy of p300 was similar among the three clusters (Figure 3C). Comparing our ChIP-seq data with H3K9ac (another activation-associated histone modification) ChIP-seq data from Ptasinska *et al* [24], we observed that enrichment of H3K9ac was inversely correlated with our AML1-ETO and N-CoR occupancies (data not shown). This inverse relationship between AML1-ETO/N-CoR and activating histone marks reinforces the idea that AML1-ETO acts as a transcriptional repressor at many loci in Kasumi-1 cells.

We examined the distribution of AML1/AML1-ETO bound loci from all three clusters among genomic elements (Figure 3B). AML1/AML1-ETO occupied regions from Clusters I and III are gene-distal compared to those of Cluster II. Yet, for each cluster the comparison with occupancy expected based upon AML1/AML1-ETO binding with random sequences or sequences that match the AML1 motif revealed differences. For example, for all clusters AML1/AML1-ETO occupancy at promoters and exons is higher than expected (Figure 3B). As noted above, the occupancy reflected by Cluster II is much more strongly associated with TSS-proximal elements than that of Clusters I and III.

The sequences with the greatest AML1-ETO/N-CoR co-occupancy (Cluster I) were compared with the remaining sequences occupied by AML1/AML1-ETO (Clusters II and III) using a discriminatory motif analysis (see Methods). The ETS/PU.1 motif was over-represented among the Cluster I sequences (p < 2.2 × 10^−16^ using a Kolmogorov-Smirnov test). The resulting PU.1 motif (Additional file 8: Figure S7) was scanned across all AML1/AML1-ETO-occupied sequences using FIMO [39], which demonstrated the relative enrichment of the PU.1 motif in distal, predominantly N-CoR-associated putative regulatory sequences (Cluster I) and depletion in the proximal transcriptionally active sequences (Cluster II) (Figure 3D). Thus, AML1-ETO/N-CoR interactions likely perturb PU.1 function at multiple loci in the Kasumi-1 cell genome, in turn contributing to the leukemic phenotype of these cells.

### The AML1-ETO/N-CoR signature is linked to AML1-ETO target gene expression and leukemia

**Figure 4:**
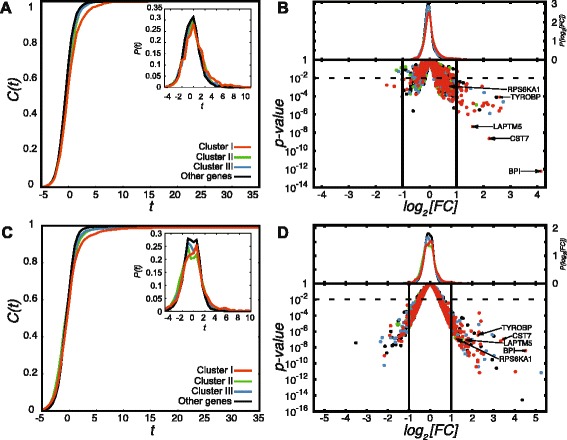
Genes associated with the AML-ETO/N-CoR signature have the greatest recovery upon AML1-ETO knockdown. **(A)** The cumulative distribution of *t* scores, $C(t)={\displaystyle {\int}_{-\infty}^tP(s)ds}$, comparing changes in gene expression for each of the clusters (Figure 3A) due to reduction in mRNA levels of the AML1-ETO fusion protein [30] is shown. For a given gene, the *t* score is the estimated log fold-change between conditions divided by its standard error: we observe that C(*t*) is shifted significantly (p < 4.6 × 10^−10^ using a t-test) to higher *t* for Cluster I compared to either Clusters II or III. The inset shows the probability distributions, *P*(*t*), for each cluster. **(B)** Volcano plot of expression differences due to RNAi mediated knockdown of AML1-ETO [30] showing genes from each of the clusters. The horizontal axis shows log_2_(fold-change), with vertical lines indicating cutoffs of 2-fold in either direction. The upper panel displays the distributions of log_2_(fold-change) for each of the clusters, again with a significant shift (p < 4 × 10^−9^ using a t-test) to higher fold-changes for Cluster I, relative to the other two clusters. **(C)** Distributions of t scores, as in **(A)** using expression profiling data collected over several time-points in Kasumi-1 cells under knockdown of the AML1-ETO mRNA [24]. We observe that C(*t*) is shifted significantly (p < 2.9 × 10^−8^ using a t-test) to higher *t* for Cluster I compared to either Clusters II or III. **(D)** Volcano plot of expression differences due to knockdown of AML1-ETO [24] showing genes from each of the clusters. The upper panel displays the distributions of log_2_(fold-change) for each of the clusters, again with a significant shift (p < 3.5 × 10^−8^ using a t-test) to higher fold-changes for Cluster I, relative to the other two clusters.

In order to relate the AML1-ETO/N-CoR co-occupancy signature to phenotypic changes in gene expression, putative regulatory target genes were interrogated using publicly available expression profiling data from AML1-ETO mRNA knockdown experiments in Kasumi-1 cells [30]. For a given gene, the *t* score is the estimated log fold-change between conditions divided by its standard error: we observe that P(*t*) is shifted significantly to higher *t* for Cluster I compared to either Clusters II or III (Figure 4A). This observation is further evident in the volcano plot (Figure 4B), where genes associated with Cluster I are over-represented (p < 2.5 × 10^−5^ comparing with either Clusters II or III using Fisher’s exact test) among genes with substantially altered expression^a^. Thus, relative to other AML1/AML1-ETO-bound genes, those with the AML1-ETO/N-CoR signature (Cluster I) were more transcriptionally repressed and therefore up-regulated in AML1-ETO depleted Kasumi-1 cells. These results were recapitulated using a second set of publicly available expression profiling data from AML1-ETO mRNA knockdown experiments in Kasumi-1 cells [24] (see Figure 4C and D). In Figure 4D, genes associated with Cluster I are over-represented (p < 2.5 × 10^−4^ comparing with either Clusters II or III using Fisher’s exact test) among genes with substantially altered expression^1^.

**Figure 5:**
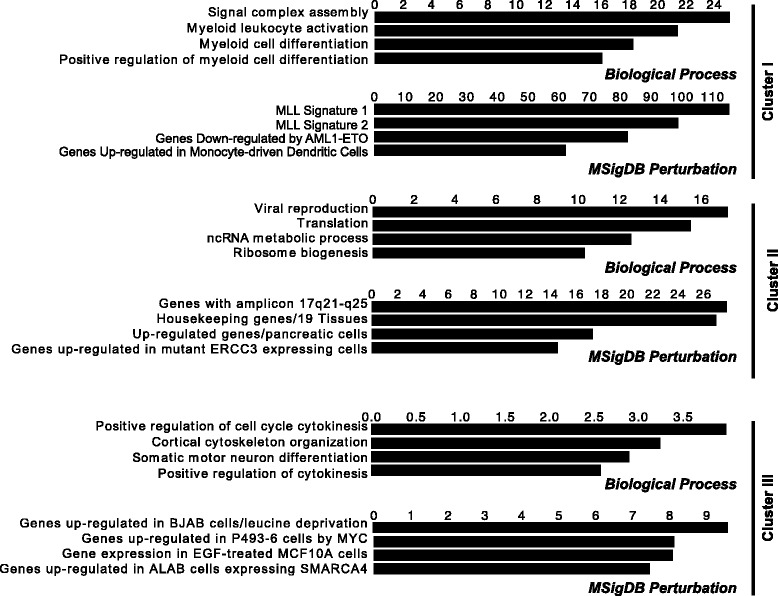
Ontologies for shared AML1-ETO and N-CoR genomic regions are relevant to leukemia. Gene ontology categories (Biological Process; Molecular Signatures Database Perturbation) with associated top-ranked terms are shown for each cluster. Values on column plots represent –log_10_(binomial p-value), computed using GREAT [35] (version 2.0.2) with the default association rules. Note: some ontology term names were shortened.

Because we have observed a clear relationship between AML1-ETO/N-CoR co-localization and siRNA-induced abrogation of the leukemic phenotype in Kasumi-1 cells (Figure 4A), it is worthwhile to compare the genes linked with different patterns of AML1/AML1-ETO/co-factor occupancy with those from gene ontology terms. We find that the AML1-ETO/N-CoR signature is associated with phenotypic terms including “myeloid cell differentiation”, while Clusters II and III are more associated with normal cellular function (Figure 5). Taken together, gene expression and ontology data suggest that the AML1-ETO/N-CoR signature establishes the leukemic phenotype.

## Discussion

Our ChIP-seq data demonstrate that AML1-ETO binding regions are more correlated with N-CoR in Kasumi-1 cells than any other assayed proteins, including p300. Using ChIP (re-ChIP) studies in Kasumi-1 cells, Ptasinska et al determined that AML1-ETO preferentially recruits the co-repressor HDAC2 (an N-CoR interacting partner) instead of the co-activator p300 [25]. This genome-wide study also revealed that AML1-ETO bound loci are chiefly associated with transcriptional repression [25]. Thus, the preferential association between AML1-ETO and N-CoR in our study supports the finding that many AML1-ETO regulated genes are repressed [24,25]. In SKNO-1 cells, another *in vitro* model system for t(8;21) leukemia, AML1-ETO and p300 were also found to co-occupy genomic regions [26], yet the relevance to the t(8;21) disease phenotpye is unclear. Our integration of ChIP-seq data with publicly available microarray data and gene ontology analyses demonstrate that regions co-occupied by AML1-ETO/N-CoR are more relevant to t(8;21) leukemia than AML1-ETO/p300.

We found that genomic regions of AML1 occupancy largely overlap AML1-ETO binding regions. A similar result has also been observed in ChIP-chip data of U937 cells over-expressing AML1-ETO [40]. Ptasinska and colleagues determined global DNA binding for AML1 and AML1-ETO in Kasumi-1 cells [24,25]. Although we used different AML1 and ETO antibodies for our ChIP experiments, there is a very high correspondence (80%) of peaks between the datasets (data not shown). Similar to reports of other transcription factors in hematopoietic cell types [41,42], AML1 and AML1-ETO largely occupy promoter-distal sites (including introns) in the Kasumi-1 genome. These results suggest that these distal sites serve as platforms for AML1-ETO to regulate transcription via long-range chromatin interactions. AML1 and fusion protein occupancy at distal sites may be important for maintaining chromatin structure and for scaffolding protein-protein interactions. Approximately 20% of AML1, fusion protein, and co-regulator peaks occupy the same genomic regions. These ubiquitous sites may reflect cell population effects and/or the different affinities of p300 and N-CoR for AML1-ETO. Alternatively, co-occupancy of opposing factors, N-CoR and p300, may be an important mechanism for fine tuning chromatin at regulatory loci [43].

ChIP-seq data have been used to identify putative protein partners via *de novo* motif discovery [41,44,45]. Each of our libraries was enriched in ETS factor motifs. A motif for one candidate ETS family member, FLI1, was discovered within significant N-CoR and p300 peaks (Figure 2C). Our data corroborate a recent report showing that FLI1 binds regions similar to AML1-ETO and that the fusion protein is recruited to ETS factor binding sites [27]. Although the AML1-ETO/N-CoR signature appears to largely account for the leukemic phenotype, Myb-p300 interactions may play a small role in this disease phenotype. The Myb motif was enriched in our p300 ChIP-seq dataset. Interestingly, Pattabiraman *et al* demonstrated that interactions between C-Myb and p300 are important for initiating acute myeloid leukemia [46]. Strikingly, ETS/PU.1 motifs were over-represented in Cluster I, implying that AML1-ETO is potentially recruited by PU.1 and may be necessary for maintaining differentiation arrest in Kasumi-1 cells. This assertion, along with the specific identity of PU.1 from among the highly similar ETS-family motifs, was confirmed by Ptasinska *et al*, who performed PU.1 ChIP-seq in Kasumi-1 cells and found a high degree of overlap between PU.1 and AML1-ETO associating regions [25]. Because AML1-ETO binds DNA and also interacts with PU.1-bound to DNA [22], the fraction of AML1-ETO peaks that represent directly versus indirectly bound DNA is not known.

Previous reports have demonstrated that depletion of AML1-ETO in Kasumi-1 cells resulted in global increases in H3K9ac occupancy, an epigenetic mark for transcriptional activation [24]. We examined genome-wide enrichment sites for H3K4me3 and H3K27me3, marks of activation and repression, respectively. AML1-ETO mediated gene repression is believed to occur through an epigenetic mechanism involving H3K27me3 [12]. Although this activity occurs at the LAT2 gene [47] and only on a subset of our ChIP-seq peaks, the repression characterized by local alterations in H3K27me3 is likely not the dominant mechanism in Kasumi-1 cells. In contrast, H3K4me3 enrichments correlated well with those of H3K9ac [24]; signals for both activating marks were reduced in genomic regions where AML1-ETO and N-CoR were elevated (data not shown). HDAC activity may decrease H3K9ac signals at these regions, a change that may be achieved by direct interactions between AML1-ETO and HDACs or via recruitment of HDACs by N-CoR. Similarly, in transcription start site-proximal regions, H3K4me3 enrichment may be reduced when AML1-ETO/N-CoR occupancy is high.

Given that the fusion protein occupies Kasumi-1 metaphase chromosomes [20], it is possible that during mitosis AML1-ETO associates with chromatin regulators at novel genes that are critical for maintaining the leukemic phenotype. This epigenetic gene bookmarking mechanism has been described for Runx2 in osteoblastic cells [33,48,49] and for PcG proteins in Drosophila S2 cells [50]. Future work on purified populations of mitotic Kasumi-1 cells will shed light on whether AML1-ETO participates in gene bookmarking.

Collectively, our data have revealed a global AML1-ETO/N-CoR signature with two key properties: occupancy of promoter-distal regions of AML1-ETO-regulated genes, and enrichment for myeloid-related ETS factors. Genes within this regulatory network define the disease phenotype of t(8;21) leukemia and are potential therapeutic targets.

## Conclusions

Findings presented here establish a novel t(8;21) AML leukemia signature characterized by occupancy of AML1-ETO/N-CoR at promoter-distal genomic regions enriched in motifs for myeloid differentiation factors. These findings are significant because a genome-wide mechanism for AML1-ETO mediated block in myeloid differentiation remains poorly understood. Results reported in this study provide insight into genome-wide mechanisms that contribute to the disease phenotype of the t(8;21) carrying leukemia.

## Methods

### Cell lines and reagents

Kasumi-1 and K562 cell lines were purchased from the American Type Culture Collection (ATCC, Manassas, VA) and maintained in RPMI-1640 media supplemented with 20% FBS. AML1 antibodies were purchased from Cell Signaling Technology, Inc. (4334, Danvers, MA), Abcam (50541, Cambridge, MA), EMD Millipore (PC285, Billerica, MA), and Active Motif (39000, Carlsbad, CA). The Cell Signaling Technology and Active Motif AML1 antibodies gave robust AML1 pulldowns and displayed significant ChIP-PCR enrichments of control genomic regions. The Cell Signaling Technology AML1 antibody recognizes the N-terminal region of AML1, thus pulling down both wildtype AML1 and AML1-ETO. In contrast, the Active Motif AML1 antibody recognizes the C-terminal region of AML1 and does not pull down the AML1-ETO fusion protein. Therefore, the Active Motif antibody was used for AML1 ChIP-seq library preparations. An ETO antibody, PC283, was purchased from EMD Millipore (Billerica, MA). N-CoR (SC-1609) and p300 (SC-585) antibodies were purchased from Santa Cruz Biotechnology, Inc. (Santa Cruz, CA). A histone H3 (tri methyl K4) antibody (ab1012) was purchased from Abcam (Cambridge, MA) and a histone H3 (tri methyl K27) antibody (ABE44) was purchased from EMD Millipore (Billerica, MA).

### ChIP-seq library preparation

Kasumi-1 cells growing in log phase were fixed with 1% formaldehyde for 10 min at room temperature and subsequently quenched with 0.25 M glycine. After washes in PBS, cells were flash frozen in liquid nitrogen. Thawed pellets were resuspended in Buffer A (50 mM HEPES, 140 mM NaCl, 1 mM EDTA, 10% glycerol, 0.5% NP-40, 0.25% Triton X-100) and rotated at 4°C for 10 min. Following centrifugation, pellets were resuspended in Buffer B (10 mM Tris-HCl, 200 mM NaCl, 1 mM EDTA, 1 mM EGTA) and rotated at 4°C for 10 min. Pellets from centrifuged samples were resuspended in Buffer C (10 mM Tris-HCl, 100 mM NaCl, 1 mM EDTA, 1 mM EGTA, 0.1% sodium deoxycholate, 0.5% N-lauroylsarcosine). Samples were aliquoted, 3 ml per tube, and sonicated to a fragment size of 100-500 base pairs using a 3.2 mm sonication probe (QSonica, Newtown, CT). Sheared chromatin (from approximately 30 million cells) was immunoprecipitated with antibodies overnight. All ChIP experiments included either normal goat or rabbit IgG (EMD Millipore, Billerica, MA) as controls. Antibody-lysate complexes were mixed with Protein G Dynabeads (Life Technologies, Grand Island, NY) for two hours. For AML1, ETO, H3K4me3, and H3K27me3 immunoprecipitations, beads were washed once with IP buffer, three times with RIPA buffer (50 mM HEPES, 500 mM LiCl, 1 mM EDTA, 0.5% NP-40, 0.25% sodium deoxycholate), once with PBS, and once with TE buffer. For N-CoR and p300 pulldowns, beads were washed once with IP buffer, once with high salt buffer (2 mM EDTA, 20 mM Tris-HCl, 500 mM NaCl), 1 time with RIPA buffer (50 mM HEPES, 250 mM LiCl, 1 mM EDTA, 0.5% NP-40, 0.25% sodium deoxycholate), once with PBS, and once with TE buffer. Protein-DNA complexes were extracted from beads at 37°C in elution buffer (10 mM EDTA, 50 mM Tris-HCl, 1% SDS). For reverse crosslinking, supernatants from centrifuged samples were rotated overnight at 60°C. RNAse and proteinase K treated samples were extracted with phenol:chloroform:isoamyl alcohol. Precipitated DNA was resuspended in 10 mM Tris-HCl, quantified with a Qubit fluorometer (Life Technologies, Grand Island, NY).

Prior to processing raw ChIP material for deep sequencing, ChIP-PCR validation studies were employed. Positive and negative control binding regions were established using different primers sets, and data was expressed as percentage of input for each region. In addition to site-specific enrichment for ChIP samples, IgG was used as the non-specific control. A second round of ChIP-PCR was performed following amplification of libraries to ensure enrichment of genomic regions in ChIP samples relative to input. Primer sequences used in ChIP-PCR studies (including ChIP-seq target validation) can be found in Additional file 9: Table S2.

The Illumina protocol (Illumina, Inc., San Diego, CA) for ChIP-seq library generation was used with slight modifications. Approximately 5-10 ng chromatin was end-repaired (EpiCentre Biotechnologies, Madison, WI). Material was then A-tailed and ligated with adapters for single end deep sequencing (Illumina, Inc., San Diego, CA). Adapter modified DNA was size-selected, 300-400 base pair (bp) range, and then amplified using the Phusion polymerase (New England Biolabs, Ipswich, MA). Amplified ChIP libraries were size selected and sequenced on an Illumina GAIIx Genome Analyzer (Illumina, Inc., San Diego, CA) at the UMass Medical School Deep Sequencing Core Facility (Worcester, MA). Two biological ChIP-seq replicates and inputs were collected for AML1, AML1-ETO, p300, N-CoR, H3K4me3, and H3K27me3.

### Analysis of sequencing data

As a preliminary step, the read quality from ChIP-seq experiments was assessed using FastQC [51]. Reads were aligned to the human genome (GRCh37, hg19) using bowtie (version 0.12.8) [52], allowing up to two mismatches. Peak calling was performed using MACS (version 2.0.10.20131216) [34] with default settings and a p < 10^−20^ threshold. For the remainder of this report, replicates for each regulatory protein or histone modification were pooled prior to peak calling and the resulting occupancy/enrichment profiles were normalized to 10 million reads. The overlaps between ChIP-seq peaks, summarized in Figure 1, were based upon peak summits ± 50 bp. Using binding loci for AML1 and/or AML1-ETO (peak summits ± 50 bp, with peaks merged from the two pooled experiments), mean ChIP-seq read densities were collected for a set of ChIP-seq experiments and clustered by k-means (k = 3). In order to determine an appropriate choice for k, we clustered our signals, examined the sum of the squared error (SSE) for 1 ≤ *k* ≤ 15 and compared this error with that from a set of 250 randomized instances of the data [53]. We observed that the difference between the actual SSE and that for randomized data was maximal for k = 3.

*De novo* motifs were detected for each of the assayed proteins (Figure 2C) using HOMER (v. 3.15) [54]. Distinguishing binding of specific transcription factors within the ETS family is difficult because ETS factor motifs are similar. Therefore, our analysis makes a conservative assignment by designating these motifs “ETS family”. Candidate ETS transcription factors that relate to t(8;21) leukemia are listed in the Discussion section. To detect a *de novo* motif that can best distinguish between sequences from Cluster I and those in Clusters II and III, we used the area under the receiver operating curve (AUC) to compare candidate motifs [55] converging from a random motif to an optimal one using a simulated annealing procedure [56] with Metropolis-Hastings Monte Carlo moves [57,58]. The resulting PU.1 *de novo* motif (Additional file 8: Figure S7) was compared to known motifs using TOMTOM (version 4.9.1) [59] and was used with FIMO [39] (version 4.7.0) with a significance threshold of p < 10^−4^ to scan sequences in the clusters.

In order to evaluate the transcriptional effect of AML1-ETO binding in association with N-CoR and p300, two sets of previously reported gene expression profiling data [24,30] were independently used in combination with our clustered ChIP sequencing data. The first published expression data that we compared with [30] were collected from Kasumi-1 cells that had been transfected with AML1-ETO or luciferase siRNA constructs by either Amaxa nucleofection or using Bio-Rad siLentFect. The raw expression data were preprocessed and normalized using GCRMA [60]. These Kasumi-1 microarrays were originally processed in two batches on different dates and the global expression patterns were strongly clustered by batch. We were able to control for this effect using the linear model “~0 + transfection + AML1-ETO”, where the “transfection” factor correlates with the batch dates and the “AML1-ETO” factor indexes knockdown (or not) of the AML1-ETO mRNA. Replicates were treated as blocking factors in the linear model using limma [61] and empirical Bayes-moderated *t* tests were performed (Figure 4A and B). The linear model was fitted only for probe-sets that were annotated with Entrez gene IDs; when there was more than one probe-set with the same gene ID, only the probe-set with the largest interquartile range was retained. A regulatory target gene can be assigned to each AML1/AML1-ETO locus on the basis of locus-TSS proximity. For each gene, however, there may be many candidate regulatory loci. Therefore, an additional assumption was made: regulation via an AML1-ETO/N-CoR (i.e., Cluster I) locus is dominant. If a gene is putatively regulated by loci from each of Clusters I, II and III, the Cluster I locus is assumed to be limiting in its control of expression and the gene is assigned exclusively to Cluster I for the purposes of comparing aggregate changes in expression due to binding for each of the clusters. A Fisher’s exact test was used to measure associations between the Clusters and genes regulated by AML1-ETO.

In order to reinforce the above analysis of transcriptional outcomes associated with AML1-ETO/N-CoR co-localization, we carried out steps similar to those outlined above using another set of previously reported gene expression profiling data, collected in Kasumi-1 cells under knockdown (or not) of the AML1-ETO mRNA [24]. These expression data were collected, without replication, over a series of four time-points following electroporation with either AML1-ETO siRNA or mismatch siRNA. Normalized expression data were collected from the NCBI Gene Expression Omnibus [62]. After retaining only probe-sets that were annotated with Entrez gene IDs and, when there was more than one probe-set with the same gene ID, retaining only the probe-set with the largest interquartile range, a linear model was fitted. We used the linear model “~0 + time-point + AML1-ETO”. The “time-point” variable enforces explicit pairing between the AML1-ETO and mismatch siRNA conditions: the resulting empirical Bayes-moderated *t* tests from limma [61] are, therefore, paired *t* tests (Figure 4C and D).

### Immunoprecipitation and western blotting

Kasumi-1 cells were centrifuged at 300 × g, resuspended in PBS, and centrifuged again at 300 × g. Cells were lysed in buffer (20 mM Tris, 0.15M NaCl, 1 mM EDTA, 1 mM EGTA, 1% Triton X-100, 1 mM PMSF), vortexed, and kept on ice for 20 min. Samples were centrifuged at 10,000 × g for 10 min. Supernatants were collected and protein concentration was determined by the BCA Assay (Thermo Scientific, Rockford, IL). Whole cell Kasumi-1 extracts (500 μg) were immunoprecipitated overnight with 2 μg of either p300 or N-CoR antibodies (Santa Cruz Biotechnologies Inc., Santa Cruz, CA). Rabbit or goat IgG were used as controls (Millipore, Billerica, MA). Samples were rotated with 30 μl Protein G Dynabeads (Life Technologies, Grand Island, NY) for two hours, and then washed five times with IP buffer. Protein samples were run on a 5% SDS-PAGE gel and transferred to PVDF membranes (Thermo Scientific, Rockford, IL). Membranes were blotted with p300 and N-CoR antibodies (1:750 dilution) overnight. Secondary antibodies conjugated with HRP were purchased from EMD Millipore (Billerica, MA). Enhanced chemiluminescence was used for protein detection (Thermo Scientific, Rockford, IL).

### Availability of supporting data

The data discussed in this publication have been deposited in NCBI’s Gene Expression Omnibus [62] and are accessible through GEO Series accession number GSE62847 (http://www.ncbi.nlm.nih.gov/geo/query/acc.cgi?acc=GSE62847).

### Endnote

^a^Genes exhibiting substantially altered expression are defined here to have log_2_(fold-change) > 1 and p < 0.01.

## Additional files

Additional file 1: Figure S1. Antibody validation prior to ChIP library preparation. (A) ChIP-western experiments on crosslinked Kasumi-1 cells using a panel of AML1 antibodies and an AML1-ETO antibody. An Active Motif AML1 antibody (39000, Carlsbad, CA) was used for ChIP-seq library preparations. Blots were cropped for clarity. (B) Western blots for p300 and N-CoR using Kasumi-1 and K562 whole cell lysates. (C) Immunoprecipitation and western blot of N-CoR in Kasumi-1 cells (using ChIP buffer C). IgG served as the control for all experiments. Additional file 2: Figure S2. ChIP library validation in Kasumi-1 cells. (A) ChIP-PCR experiments demonstrated significant pulldown of a region in the Runx1P1 promoter in ChIP samples but not in IgG control samples. Binding was negligible at the negative control hPhox region. The strength of pulldowns are expressed as percent input. Experiments were repeated twice and error bars represent standard deviation. (B) Representative bioanalyzer results for AML1 and AML1-ETO libraries. Each library displays a size-selected, narrow fragment range amenable for deep sequencing. Additional file 3: Table S1. ChIP-Seq library overview. Shown are the number of reads and peaks called for each replicate ChIP-Seq library. Additional file 4: Figure S3. Tag density plots and ChIP validaton of AML1-ETO target genes. (A) Tag density plots displaying enriched AML1-ETO regions corresponding to known fusion protein target genes. Asterisks indicate the positions of TYROBP, LAPTM5, and RPS6KA1 regions validated in ChIP-PCR studies (B). Fold enrichment indicates enrichment of ChIP samples over inputs when equivalent amounts of DNA were used in PCR reactions. Additional file 5: Figure S4. Correlation between different ChIP-seq libraries. Scatter plots for five ChIP-seq experiments collected using Kasumi-1 cells. Each point represents a region of enrichment (i.e. “peak”) in either the AML1, AML1-ETO or both experiments, with the normalized mean read number, *S*_*X*_, plotted for five antibodies with target *X* on a logarithmic scale. The lower triangle of the figure is comprised of scatter plots for pair-wise comparisons, while the upper triangle reports the corresponding correlation coefficients (Pearson’s r). Additional file 6: Figure S5. Ontologies for genomic regions co-occupied by AML1-ETO and co-regulatory proteins. (A-B) Gene ontology (GO) categories (Biological Process; Molecular Signatures Database Perturbation) with associated top-ranked terms are shown for genomic regions associated with exclusive AML1-ETO/N-CoR enrichments (see 4252 overlapping regions in Figure 1C) and AML1-ETO/p300 enrichments (see 1164 overlapping regions in Figure 1C). These genomic regions were defined using the Venn diagram in Figure 1C and genomic coordinates were associated with GO categories using GREAT [35] (version 2.0.2). Note that no Biological Process terms were reported by GREAT (using the default association rules) for AML1-ETO/p300. Ontology terms reflecting regions of shared occupancy (see Figure 1C) between AML1-ETO/N-CoR/p300 (C) and AML1/N-CoR/p300 (D) are also reported. Values on column plots represent –log_10_(binomial p-value), computed using GREAT with the default association rules. Note: some ontology term names were shortened. Additional file 7: Figure S6. H3K27me3 ChIP-seq regions. Tag density plots of H3K27me3 data (normalized to 10^7^ reads) at CXCR4 and HCK loci. Additional file 8: Figure S7.
*De novo* discriminative motif. *De novo* motif (see Methods for details) to distinguish Cluster I loci from those of the other two clusters (see Figure 3A and D). Using TOMTOM (version 4.9.1) [59] to compare this motif with those from the TRANSFAC (Matys V et al., Nucleic Acids Res. 2006, 34(Database Issue):D108-10) and JASPAR (Mathelier A et al., Nucleic Acids Res. 2014, 42(Database Issue):D142-47) repositories, the TRANSFAC V_PU1_Q4 motif was the closest match, followed by V_PU1_01 and the JASPAR motif MA0080.2 (SPI1, also known as PU.1). Additional file 9: Table S2. Oligonucleotides used in ChIP-PCR validation studies.

## Abbreviations

TSSs: Transcriptional start sites
AML: Acute myeloid leukemia
ChIP-Seq: Chromatin immunoprecipitation with high-throughput sequencing
ETO: Eight twenty-one
GREAT: Genomic regions enrichment of annotations tool
miRs: microRNAs
N-CoR: Nuclear co-repressor
NHR: *Nervy* homology regions
RHD: Runt homology domain
SMRT: *Silencing* mediator of retinoic acid and thyroid hormone receptor
t(8;21): Translocation between chromosomes 8 and 21

## References

[1] Look AT (1997). Oncogenic transcription factors in the human acute leukemias. Science.

[2] Erickson P, Gao J, Chang KS, Look T, Whisenant E, Raimondi S (1992). Identification of breakpoints in t(8;21) acute myelogenous leukemia and isolation of a fusion transcript, AML1/ETO, with similarity to Drosophila segmentation gene, runt. Blood.

[3] Miyoshi H, Shimizu K, Kozu T, Maseki N, Kaneko Y, Ohki M (1991). t(8;21) breakpoints on chromosome 21 in acute myeloid leukemia are clustered within a limited region of a single gene, AML1. Proc Natl Acad Sci U S A.

[4] Davis JN, McGhee L, Meyers S (2003). The ETO (MTG8) gene family. Gene.

[5] Meyers S, Lenny N, Hiebert SW (1995). The t(8;21) fusion protein interferes with AML-1B-dependent transcriptional activation. Mol Cell Biol.

[6] Gelmetti V, Zhang J, Fanelli M, Minucci S, Pelicci PG, Lazar MA (1998). Aberrant recruitment of the nuclear receptor corepressor-histone deacetylase complex by the acute myeloid leukemia fusion partner ETO. Mol Cell Biol.

[7] Wang J, Hoshino T, Redner RL, Kajigaya S, Liu JM (1998). ETO, fusion partner in t(8;21) acute myeloid leukemia, represses transcription by interaction with the human N-CoR/mSin3/HDAC1 complex. Proc Natl Acad Sci U S A.

[8] Gottlicher M, Minucci S, Zhu P, Kramer OH, Schimpf A, Giavara S (2001). Valproic acid defines a novel class of HDAC inhibitors inducing differentiation of transformed cells. EMBO J.

[9] Liu S, Klisovic RB, Vukosavljevic T, Yu J, Paschka P, Huynh L (2007). Targeting AML1/ETO-histone deacetylase repressor complex: a novel mechanism for valproic acid-mediated gene expression and cellular differentiation in AML1/ETO-positive acute myeloid leukemia cells. J Pharmacol Exp Ther.

[10] Zapotocky M, Mejstrikova E, Smetana K, Stary J, Trka J, Starkova J (2012). Valproic acid triggers differentiation and apoptosis in AML1/ETO-positive leukemic cells specifically. Cancer Lett.

[11] Buchi F, Masala E, Rossi A, Valencia A, Spinelli E, Sanna A (2014). Redistribution of H3K27me3 and acetylated histone H4 upon exposure to azacitidine and decitabine results in de-repression of the AML1/ETO target gene IL3. Epigenetics.

[12] Chen J, Odenike O, Rowley JD (2010). Leukaemogenesis: more than mutant genes. Nat Rev Cancer.

[13] Klampfer L, Zhang J, Zelenetz AO, Uchida H, Nimer SD (1996). The AML1/ETO fusion protein activates transcription of BCL-2. Proc Natl Acad Sci U S A.

[14] Peterson LF, Yan M, Zhang DE (2007). The p21Waf1 pathway is involved in blocking leukemogenesis by the t(8;21) fusion protein AML1-ETO. Blood.

[15] Zaidi SK, Dowdy CR, van Wijnen AJ, Lian JB, Raza A, Stein JL (2009). Altered Runx1 subnuclear targeting enhances myeloid cell proliferation and blocks differentiation by activating a miR-24/MKP-7/MAPK network. Cancer Res.

[16] Wang L, Gural A, Sun XJ, Zhao X, Perna F, Huang G (2011). The leukemogenicity of AML1-ETO is dependent on site-specific lysine acetylation. Science.

[17] Fazi F, Racanicchi S, Zardo G, Starnes LM, Mancini M, Travaglini L (2007). Epigenetic silencing of the myelopoiesis regulator microRNA-223 by the AML1/ETO oncoprotein. Cancer Cell.

[18] Liddiard K, Hills R, Burnett AK, Darley RL, Tonks A (2010). OGG1 is a novel prognostic indicator in acute myeloid leukaemia. Oncogene.

[19] Reikvam H, Hatfield KJ, Kittang AO, Hovland R, Bruserud O (2011). Acute myeloid leukemia with the t(8;21) translocation: clinical consequences and biological implications. J Biomed Biotechnol.

[20] Bakshi R, Zaidi SK, Pande S, Hassan MQ, Young DW, Montecino M (2008). The leukemogenic t(8;21) fusion protein AML1-ETO controls rRNA genes and associates with nucleolar-organizing regions at mitotic chromosomes. J Cell Sci.

[21] Lausen J, Liu S, Fliegauf M, Lubbert M, Werner MH (2006). ELA2 is regulated by hematopoietic transcription factors, but not repressed by AML1-ETO. Oncogene.

[22] Vangala RK, Heiss-Neumann MS, Rangatia JS, Singh SM, Schoch C, Tenen DG (2003). The myeloid master regulator transcription factor PU.1 is inactivated by AML1-ETO in t(8;21) myeloid leukemia. Blood.

[23] Zhang J, Kalkum M, Yamamura S, Chait BT, Roeder RG (2004). E protein silencing by the leukemogenic AML1-ETO fusion protein. Science.

[24] Ptasinska A, Assi SA, Mannari D, James SR, Williamson D, Dunne J (2012). Depletion of RUNX1/ETO in t(8;21) AML cells leads to genome-wide changes in chromatin structure and transcription factor binding. Leukemia.

[25] Ptasinska A, Assi SA, Martinez-Soria N, Imperato MR, Piper J, Cauchy P (2014). Identification of a dynamic core transcriptional network in t(8;21) AML that regulates differentiation block and self-renewal. Cell Rep.

[26] Saeed S, Logie C, Francoijs KJ, Frige G, Romanenghi M, Nielsen FG (2012). Chromatin accessibility, p300, and histone acetylation define PML-RARalpha and AML1-ETO binding sites in acute myeloid leukemia. Blood.

[27] Martens JH, Mandoli A, Simmer F, Wierenga BJ, Saeed S, Singh AA (2012). ERG and FLI1 binding sites demarcate targets for aberrant epigenetic regulation by AML1-ETO in acute myeloid leukemia. Blood.

[28] Robertson G, Hirst M, Bainbridge M, Bilenky M, Zhao Y, Zeng T (2007). Genome-wide profiles of STAT1 DNA association using chromatin immunoprecipitation and massively parallel sequencing. Nat Methods.

[29] Asou H, Tashiro S, Hamamoto K, Otsuji A, Kita K, Kamada N (1991). Establishment of a human acute myeloid leukemia cell line (Kasumi-1) with 8;21 chromosome translocation. Blood.

[30] Corsello SM, Roti G, Ross KN, Chow KT, Galinsky I, DeAngelo DJ (2009). Identification of AML1-ETO modulators by chemical genomics. Blood.

[31] Hyde RK, Liu PP (2010). RUNX1 repression-independent mechanisms of leukemogenesis by fusion genes CBFB-MYH11 and AML1-ETO (RUNX1-RUNX1T1). J Cell Biochem.

[32] van der Deen M, Akech J, Lapointe D, Gupta S, Young DW, Montecino MA (2012). Genomic promoter occupancy of runt-related transcription factor RUNX2 in Osteosarcoma cells identifies genes involved in cell adhesion and motility. J Biol Chem.

[33] Young DW, Hassan MQ, Yang XQ, Galindo M, Javed A, Zaidi SK (2007). Mitotic retention of gene expression patterns by the cell fate-determining transcription factor Runx2. Proc Natl Acad Sci U S A.

[34] Zhang Y, Liu T, Meyer CA, Eeckhoute J, Johnson DS, Bernstein BE (2008). Model-based analysis of ChIP-Seq (MACS). Genome Biol.

[35] McLean CY, Bristor D, Hiller M, Clarke SL, Schaar BT, Lowe CB (2010). GREAT improves functional interpretation of cis-regulatory regions. Nat Biotechnol.

[36] Wilson NK, Foster SD, Wang X, Knezevic K, Schutte J, Kaimakis P (2010). Combinatorial transcriptional control in blood stem/progenitor cells: genome-wide analysis of ten major transcriptional regulators. Cell Stem Cell.

[37] Hsu F, Kent WJ, Clawson H, Kuhn RM, Diekhans M, Haussler D (2006). The UCSC known genes. Bioinformatics.

[38] Bernstein BE, Birney E, Dunham I, Green ED, Gunter C, Snyder M (2012). An integrated encyclopedia of DNA elements in the human genome. Nature.

[39] Grant CE, Bailey TL, Noble WS (2011). FIMO: scanning for occurrences of a given motif. Bioinformatics.

[40] Gardini A, Cesaroni M, Luzi L, Okumura AJ, Biggs JR, Minardi SP (2008). AML1/ETO oncoprotein is directed to AML1 binding regions and co-localizes with AML1 and HEB on its targets. PLoS Genet.

[41] Pencovich N, Jaschek R, Tanay A, Groner Y (2011). Dynamic combinatorial interactions of RUNX1 and cooperating partners regulates megakaryocytic differentiation in cell line models. Blood.

[42] Tijssen MR, Cvejic A, Joshi A, Hannah RL, Ferreira R, Forrai A (2011). Genome-wide analysis of simultaneous GATA1/2, RUNX1, FLI1, and SCL binding in megakaryocytes identifies hematopoietic regulators. Dev Cell.

[43] Ram O, Goren A, Amit I, Shoresh N, Yosef N, Ernst J (2011). Combinatorial patterning of chromatin regulators uncovered by genome-wide location analysis in human cells. Cell.

[44] Bailey TL, Elkan C (1994). Fitting a mixture model by expectation maximization to discover motifs in biopolymers. Proc Int Conf Intell Syst Mol Biol.

[45] Mikkelsen TS, Xu Z, Zhang X, Wang L, Gimble JM, Lander ES (2010). Comparative epigenomic analysis of murine and human adipogenesis. Cell.

[46] Pattabiraman DR, McGirr C, Shakhbazov K, Barbier V, Krishnan K, Mukhopadhyay P (2014). Interaction of c-Myb with p300 is required for the induction of acute myeloid leukemia (AML) by human AML oncogenes. Blood.

[47] Duque-Afonso J, Yalcin A, Berg T, Abdelkarim M, Heidenreich O, Lubbert M (2011). The HDAC class I-specific inhibitor entinostat (MS-275) effectively relieves epigenetic silencing of the LAT2 gene mediated by AML1/ETO. Oncogene.

[48] Kadauke S, Udugama MI, Pawlicki JM, Achtman JC, Jain DP, Cheng Y (2012). Tissue-specific mitotic bookmarking by hematopoietic transcription factor GATA1. Cell.

[49] Zaidi SK, Young DW, Montecino M, Lian JB, Stein JL, van Wijnen AJ (2010). Architectural epigenetics: mitotic retention of mammalian transcriptional regulatory information. Mol Cell Biol.

[50] Follmer NE, Wani AH, Francis NJ (2012). A polycomb group protein is retained at specific sites on chromatin in mitosis. PLoS Genet.

[51] FastQC. [http://www.bioinformatics.babraham.ac.uk/projects/fastqc/]

[52] Langmead B, Trapnell C, Pop M, Salzberg SL (2009). Ultrafast and memory-efficient alignment of short DNA sequences to the human genome. Genome Biol.

[53] R Script for K-means cluster analysis. [http://www.mattpeeples.net/kmeans.html]

[54] Heinz S, Benner C, Spann N, Bertolino E, Lin YC, Laslo P (2010). Simple combinations of lineage-determining transcription factors prime cis-regulatory elements required for macrophage and B cell identities. Mol Cell.

[55] Whitfield TW, Wang J, Collins PJ, Partridge EC, Aldred SF, Trinklein ND (2012). Functional analysis of transcription factor binding sites in human promoters. Genome Biol.

[56] Kirkpatrick S, Gelatt CD, Vecchi MP (1983). Optimization by simulated annealing. Science.

[57] Hastings WK (1970). Monte Carlo sampling methods using Markov chains and their applications. Biometrika.

[58] Metropolis N, Rosenbluth AW, Rosenbluth MN, Teller AN, Teller E (1953). Equation of state calculations by fast computing machines. J Chem Phys.

[59] Gupta S, Stamatoyannopoulos JA, Bailey TL, Noble WS (2007). Quantifying similarity between motifs. Genome Biol.

[60] Wu Z, Irizarry RA (2004). Preprocessing of oligonucleotide array data. Nat Biotechnol.

[61] Smyth GK. Linear models and empirical bayes methods for assessing differential expression in microarray experiments. Stat Appl Genet Mol Biol.2004;3. Article 3. 10.2202/1544-6115.1027 16646809

[62] Edgar R, Domrachev M, Lash AE (2002). Gene expression Omnibus: NCBI gene expression and hybridization array data repository. Nucleic Acids Res.

[63] Kent WJ, Sugnet CW, Furey TS, Roskin KM, Pringle TH, Zahler AM (2002). The human genome browser at UCSC. Genome Res.

[64] Subramanian A, Tamayo P, Mootha VK, Mukherjee S, Ebert BL, Gillette MA (2005). Gene set enrichment analysis: a knowledge-based approach for interpreting genome-wide expression profiles. Proc Natl Acad Sci U S A.

