# Supplementary figures and images for: Genome-wide co-occupancy of AML1-ETO and N-CoR defines the t(8;21) AML signature in leukemic cells
Source: BMC Genomics. 2015 Apr 17;16(1):309. doi: 10.1186/s12864-015-1445-0 (PMC4434520; doi:10.1186/s12864-015-1445-0)

**A.**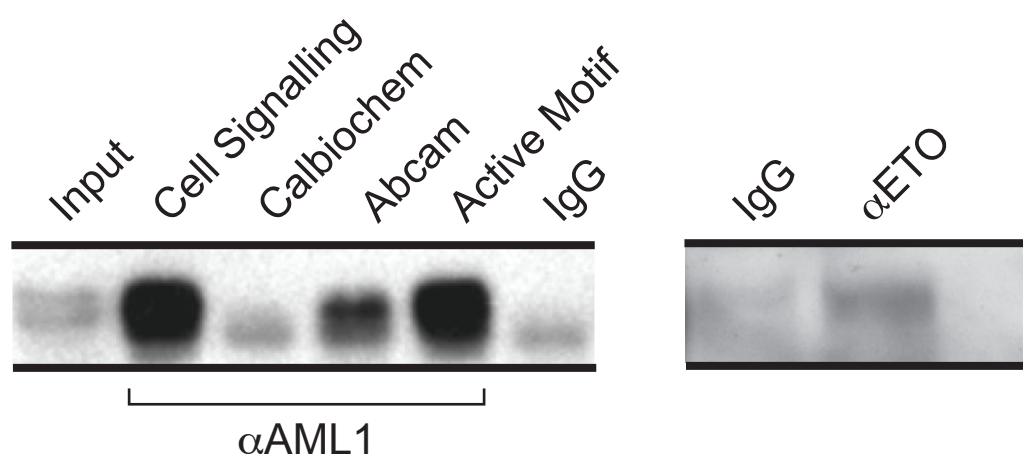**B.**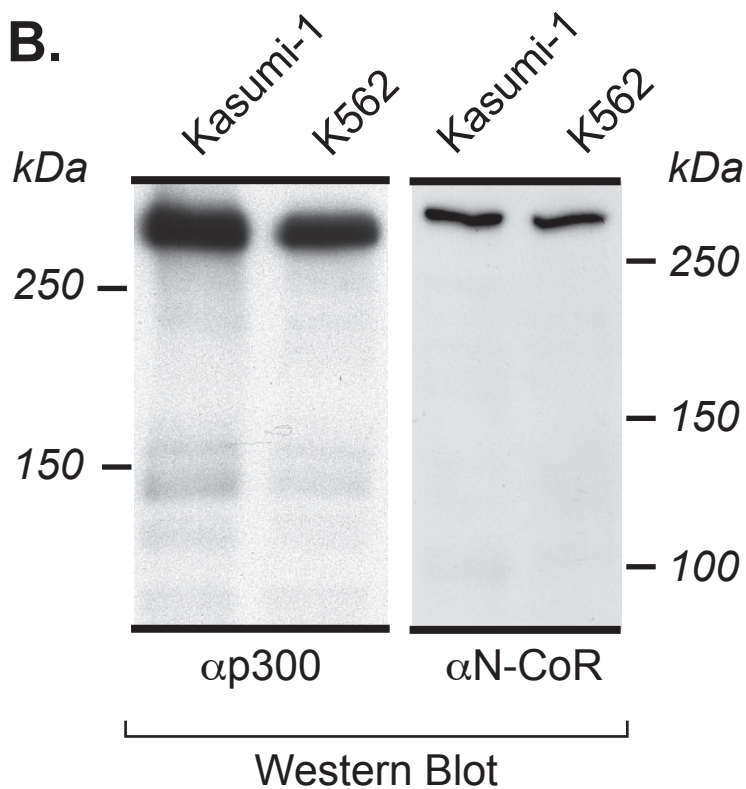**C.**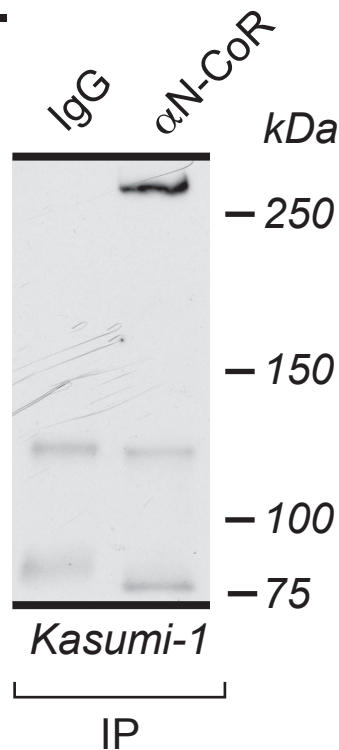

Supplement: Additional file 1: Figure S1. — Antibody validation prior to ChIP library preparation. (A) ChIP-western experiments on crosslinked Kasumi-1 cells using a panel of AML1 antibodies and an AML1-ETO antibody. An Active Motif AML1 antibody (39000, Carlsbad, CA) was used for ChIP-seq library preparations. Blots were cropped for clarity. (B) Western blots for p300 and N-CoR using Kasumi-1 and K562 whole cell lysates. (C) Immunoprecipitation and western blot of N-CoR in Kasumi-1 cells (using ChIP buffer C). IgG served as the control for all experiments. [file 12864_2015_1445_MOESM1_ESM.pdf]

**A.**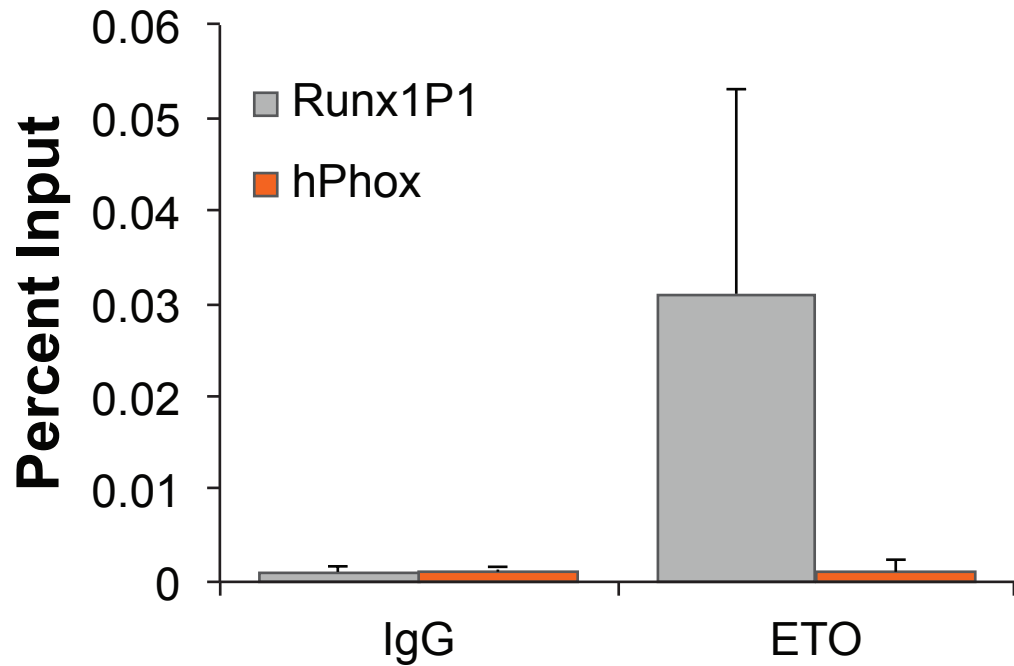**B.**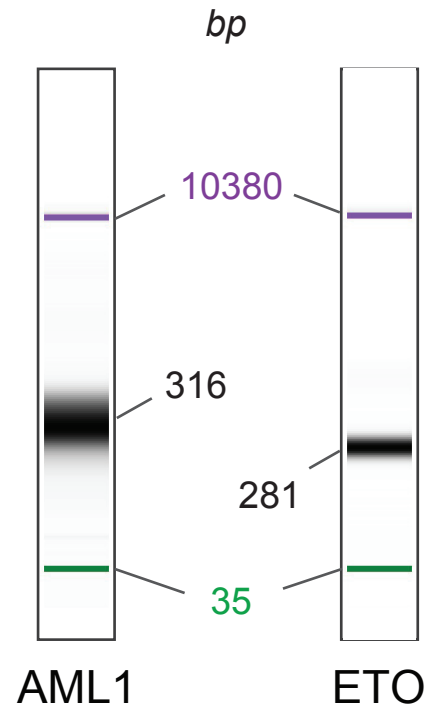

Supplement: Additional file 2: Figure S2. — ChIP library validation in Kasumi-1 cells. (A) ChIP-PCR experiments demonstrated significant pulldown of a region in the Runx1P1 promoter in ChIP samples but not in IgG control samples. Binding was negligible at the negative control hPhox region. The strength of pulldowns are expressed as percent input. Experiments were repeated twice and error bars represent standard deviation. (B) Representative bioanalyzer results for AML1 and AML1-ETO libraries. Each library displays a size-selected, narrow fragment range amenable for deep sequencing. [file 12864_2015_1445_MOESM2_ESM.pdf]

**A.***TYROBP*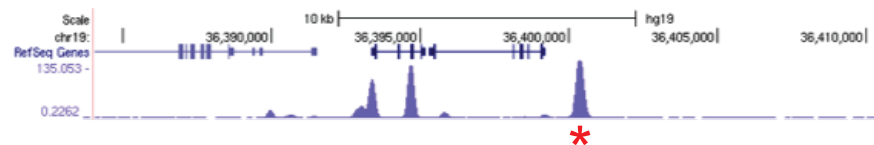*LAPTM5*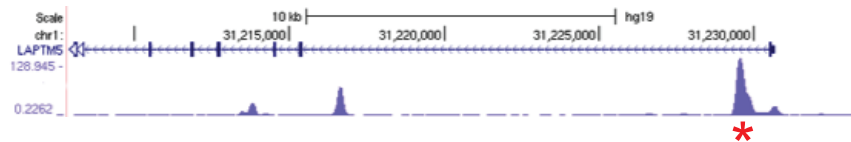*RPS6KA1*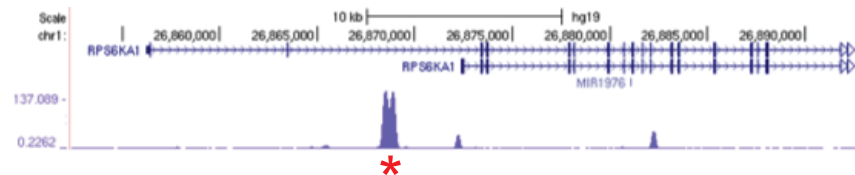**B.****Fold Enrichment**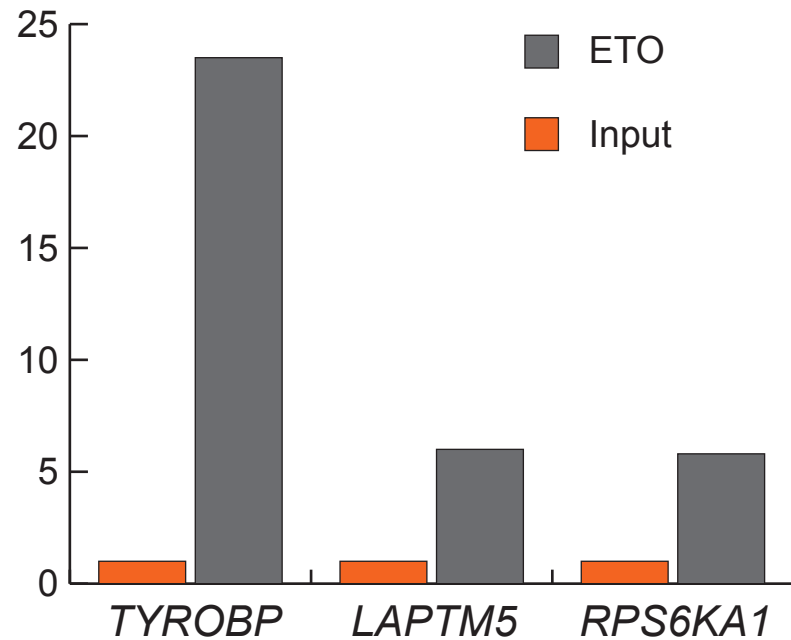

Supplement: Additional file 4: Figure S3. — Tag density plots and ChIP validaton of AML1-ETO target genes. (A) Tag density plots displaying enriched AML1-ETO regions corresponding to known fusion protein target genes. Asterisks indicate the positions of TYROBP, LAPTM5, and RPS6KA1 regions validated in ChIP-PCR studies (B). Fold enrichment indicates enrichment of ChIP samples over inputs when equivalent amounts of DNA were used in PCR reactions. [file 12864_2015_1445_MOESM4_ESM.pdf]

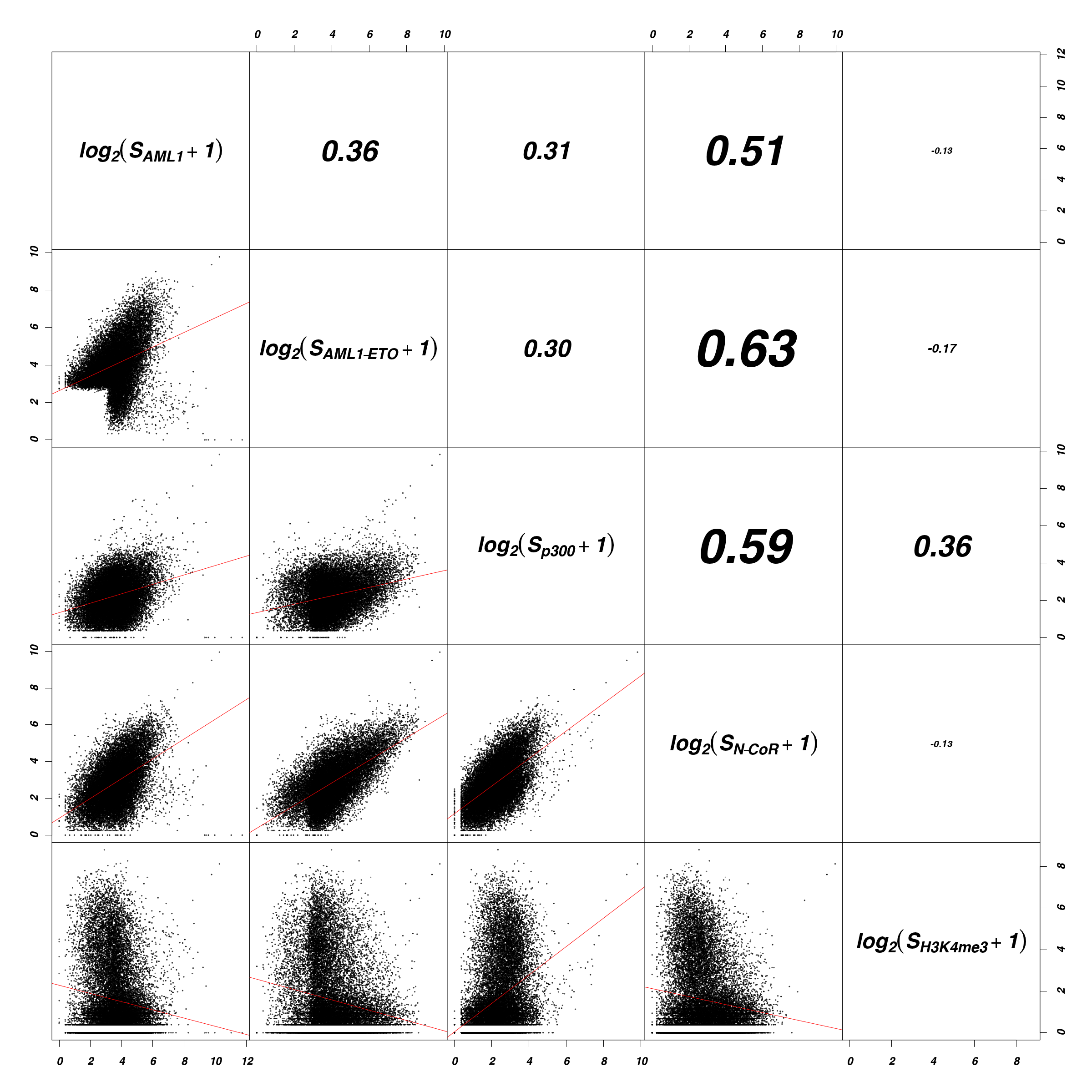

Supplement: Additional file 5: Figure S4. — Correlation between different ChIP-seq libraries. Scatter plots for five ChIP-seq experiments collected using Kasumi-1 cells. Each point represents a region of enrichment (i.e. “peak”) in either the AML1, AML1-ETO or both experiments, with the normalized mean read number, S X, plotted for five antibodies with target X on a logarithmic scale. The lower triangle of the figure is comprised of scatter plots for pair-wise comparisons, while the upper triangle reports the corresponding correlation coefficients (Pearson’s r). [file 12864_2015_1445_MOESM5_ESM.png]

## A. AML1-ETO/N-CoR

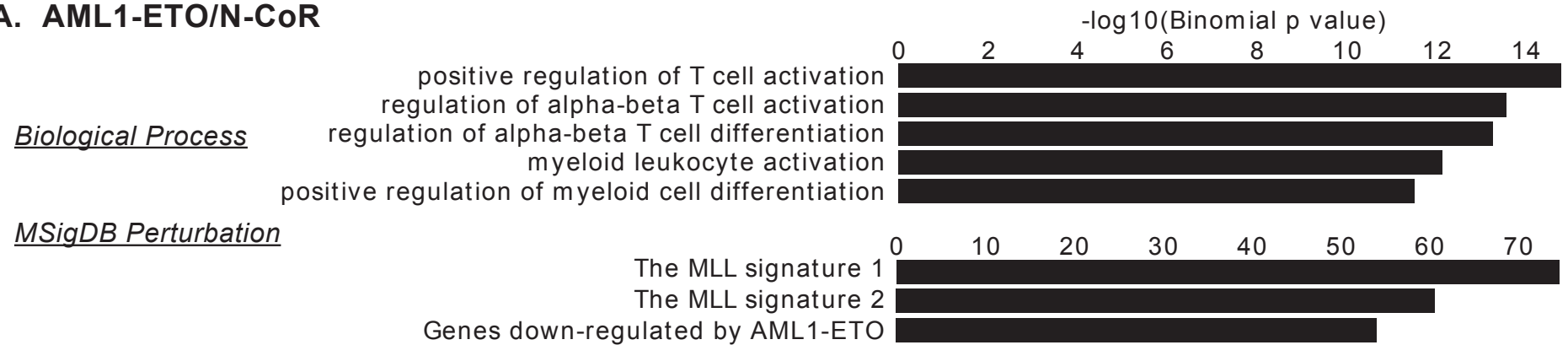

## B. AML1-ETO/p300

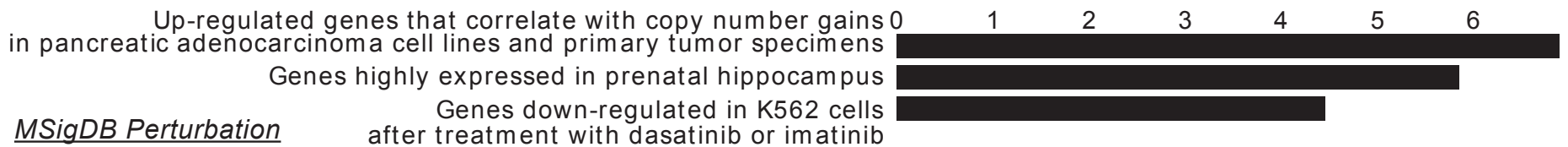

## C. AML1-ETO/N-CoR/p300

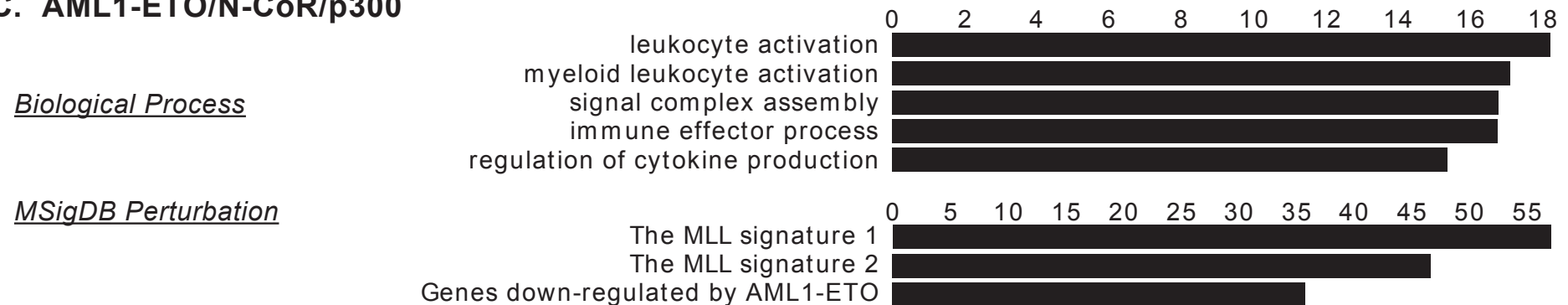

## D. AML1/N-CoR/p300

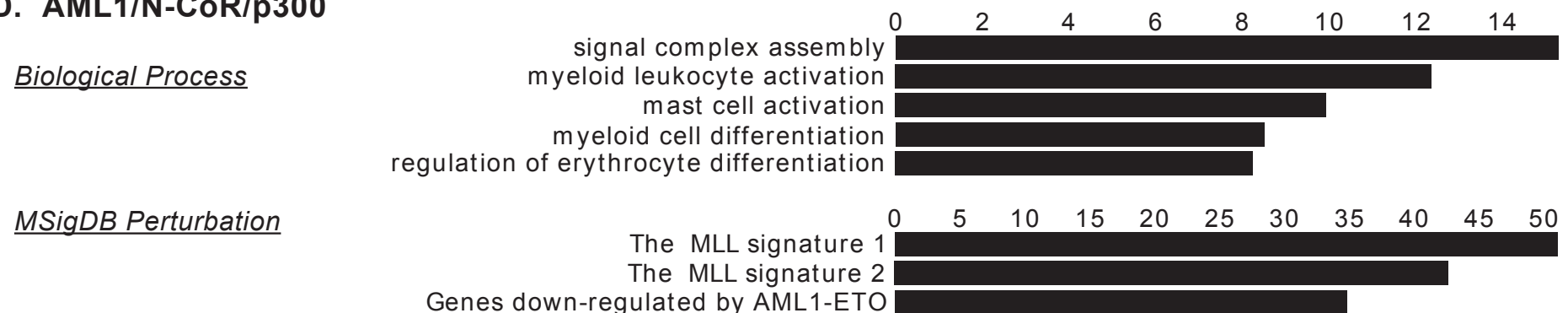

Supplement: Additional file 6: Figure S5. — Ontologies for genomic regions co-occupied by AML1-ETO and co-regulatory proteins. (A-B) Gene ontology (GO) categories (Biological Process; Molecular Signatures Database Perturbation) with associated top-ranked terms are shown for genomic regions associated with exclusive AML1-ETO/N-CoR enrichments (see 4252 overlapping regions in Figure 1C) and AML1-ETO/p300 enrichments (see 1164 overlapping regions in Figure 1C). These genomic regions were defined using the Venn diagram in Figure 1C and genomic coordinates were associated with GO categories using GREAT [35] (version 2.0.2). Note that no Biological Process terms were reported by GREAT (using the default association rules) for AML1-ETO/p300. Ontology terms reflecting regions of shared occupancy (see Figure 1C) between AML1-ETO/N-CoR/p300 (C) and AML1/N-CoR/p300 (D) are also reported. Values on column plots represent –log10(binomial p-value), computed using GREAT with the default association rules. Note: some ontology term names were shortened. [file 12864_2015_1445_MOESM6_ESM.pdf]

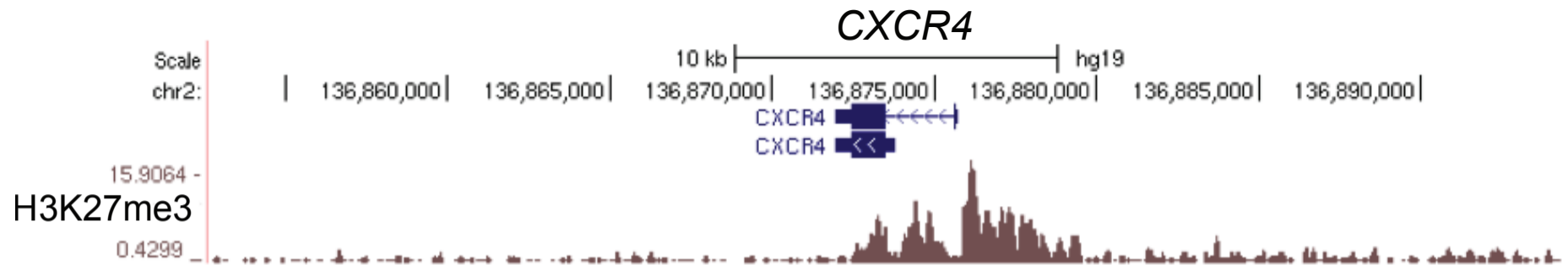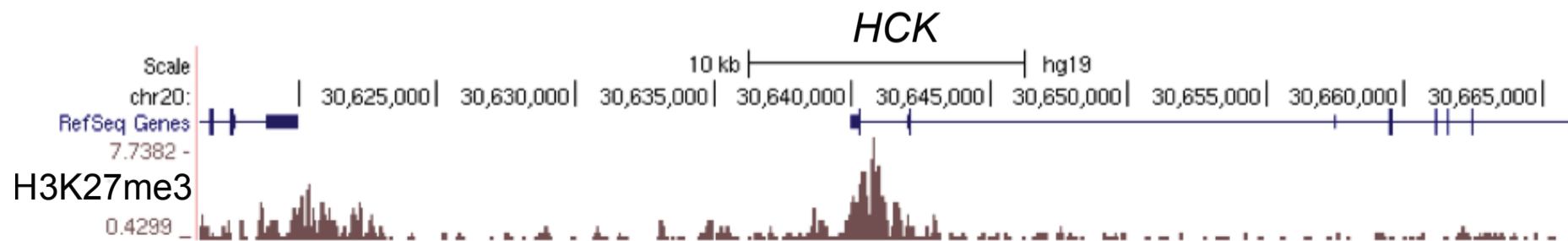

Supplement: Additional file 7: Figure S6. — H3K27me3 ChIP-seq regions. Tag density plots of H3K27me3 data (normalized to 107 reads) at CXCR4 and HCK loci. [file 12864_2015_1445_MOESM7_ESM.pdf]

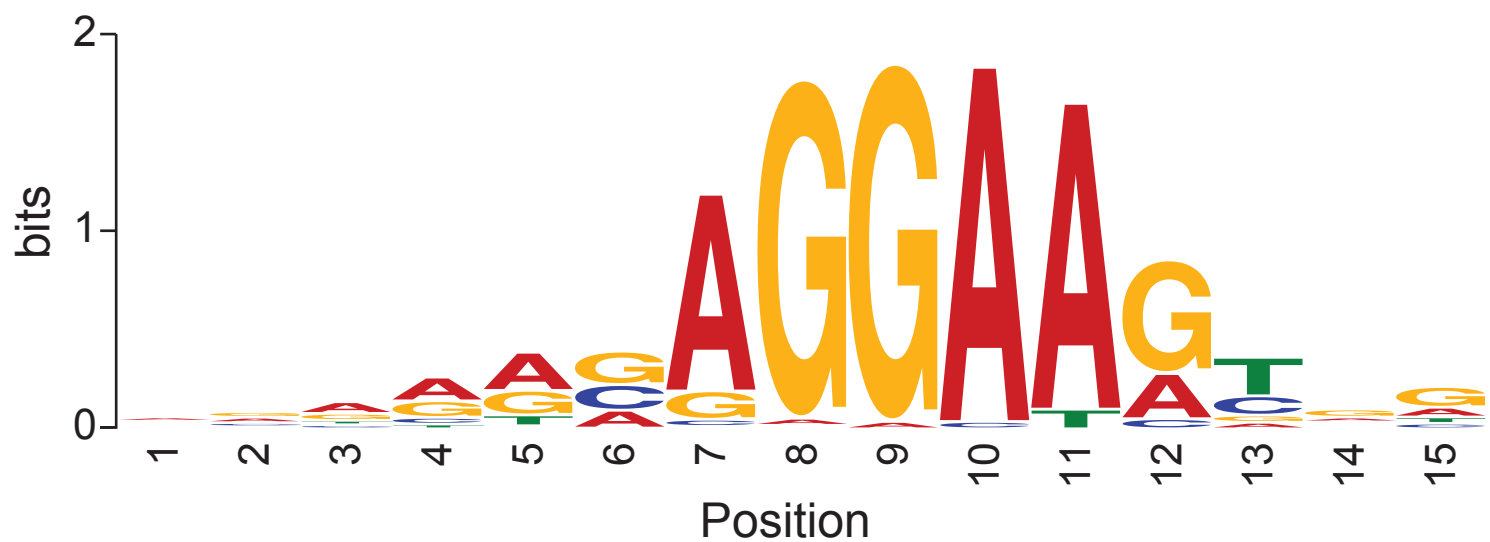

Supplement: Additional file 8: Figure S7. — De novo discriminative motif. De novo motif (see Methods for details) to distinguish Cluster I loci from those of the other two clusters (see Figure 3A and D). Using TOMTOM (version 4.9.1) [59] to compare this motif with those from the TRANSFAC (Matys V et al., Nucleic Acids Res. 2006, 34(Database Issue):D108-10) and JASPAR (Mathelier A et al., Nucleic Acids Res. 2014, 42(Database Issue):D142-47) repositories, the TRANSFAC V_PU1_Q4 motif was the closest match, followed by V_PU1_01 and the JASPAR motif MA0080.2 (SPI1, also known as PU.1). [file 12864_2015_1445_MOESM8_ESM.pdf]
