# Supplementary material for: Genome-wide co-occupancy of AML1-ETO and N-CoR defines the t(8;21) AML signature in leukemic cells
Source: BMC Genomics. 2015 Apr 17;16(1):309. doi: 10.1186/s12864-015-1445-0 (PMC4434520; doi:10.1186/s12864-015-1445-0)
Supplement: Additional file 3: Table S1. — ChIP-Seq library overview. Shown are the number of reads and peaks called for each replicate ChIP-Seq library. [file 12864_2015_1445_MOESM3_ESM.pdf]

Table S1 ChIP-Seq library overview. Shown are the number of reads and peaks called for each replicate ChIP-Seq library.

ChIP library	Million reads	No. Peaks[a]	No. Peaks[b]	No. Peaks[c]
AML1 (rep 1)	19	23,611	11,692	1,849
AML1 (rep 2)	22			
AML1-ETO (rep 1)	24	30,911	18,087	5,879
AML1-ETO (rep 2)	22			
N-CoR (rep 1)	34	21,163	10,004	1,806
N-CoR (rep 2)	28			
p300 (rep 1)	23	25,301	11,020	361
p300 (rep 2)	18			
H3K4me3 (rep 1)	18	22,386	19,632	361
H3K4me3 (rep 2)	18			
H3K27me3 (rep 1)	10	NA	NA	NA
H3K27me3 (rep 2)	14			

a. ($p < 10^{-10}$)
b. ($p < 10^{-20}$)
c. ($p < 10^{-75}$)
