# Supplementary material for: Genome-wide co-occupancy of AML1-ETO and N-CoR defines the t(8;21) AML signature in leukemic cells
Source: BMC Genomics. 2015 Apr 17;16(1):309. doi: 10.1186/s12864-015-1445-0 (PMC4434520; doi:10.1186/s12864-015-1445-0)
Supplement: Additional file 9: Table S2. — Oligonucleotides used in ChIP-PCR validation studies. [file 12864_2015_1445_MOESM9_ESM.pdf]

Table S2 Oligonucleotides used in ChIP-PCR validation studies.

Locus	Oligo (sense/antisense) 5’-3’
Runx1P1	CTGTGGGTTGGTGATGCTC<br>AGCCTGGCAGTGTCAGAAGT
Phox (cybb)	CCAATGATTATTAGCCAATTTCTG<br>CATGGTGGCAGAGGTTGAATGT
RPS6KA1	AGGCAGGAACTGAGTCATCC<br>ATGCCTTCTGACTCCTGAGC
TYROPB	AGGAGACGGGACTTCTAGC<br>TCATCCTCCAGCACCCTAAC
LAPTM5	GAGGAAGCCCTTCCAGACTC<br>GCCTAGACCCACCCTACTCC
